# In vitro antiretroviral activity and in vivo toxicity of the potential topical microbicide copper phthalocyanine sulfate

**DOI:** 10.1186/s12985-015-0358-5

**Published:** 2015-08-30

**Authors:** Ashley R. Styczynski, Khandaker N. Anwar, Habiba Sultana, Abdelhamid Ghanem, Nell Lurain, Aishi Chua, Mahmood Ghassemi, Richard M. Novak

**Affiliations:** Department of Infectious Disease, University of Illinois at Chicago, Chicago, IL 60612 USA; Department of Internal Medicine, George Washington University, Washington, DC 20037 USA; Department of Infectious Disease, Rush University, Chicago, IL 60612 USA; 755 North Ave NE #1319, Atlanta, GA 30306 USA

## Abstract

**Background:**

Copper has antimicrobial properties and has been studied for its activity against viruses, including HIV. Copper complexed within a phthalocyanine ring, forming copper (II) phthalocyanine sulfate (CuPcS), may have a role in microbicide development when used intravaginally.

**Methods:**

CuPcS toxicity was tested against cervical epithelial cells, TZM-BL cells, peripheral blood mononuclear cells (PBMC), and cervical explant tissues using cell viability assays. In vivo toxicity was assessed following intravaginal administration of CuPcS in female BALB/C mice and measured using a standardized histology grading system on reproductive tract tissues. Efficacy studies for preventing infection with HIV in the presence of various non-toxic concentrations of CuPcS were carried out in TZM-BL, PBMC, and cervical explant cultures using HIV-1_BAL_ and various pseudovirus subtypes. Non-linear regression was applied to the data to determine the EC50/90 and CC50/90.

**Results:**

CuPcS demonstrated inhibition of HIV infection in PBMCs at concentrations that were non-toxic in cervical epithelial cells and PBMCs with EC50 values of approximately 50 μg/mL. Reproductive tract tissue analysis revealed no toxicity at 100 mg/mL. Human cervical explant tissues challenged with HIV in the presence of CuPcS also revealed a dose–response effect at preventing HIV infection at non-toxic concentrations with an EC50 value of 65 μg/mL.

**Conclusion:**

These results suggest that CuPcS may be useful as a topical microbicide in concentrations that can be achieved in the female genital tract.

**Electronic supplementary material:**

The online version of this article (doi:10.1186/s12985-015-0358-5) contains supplementary material, which is available to authorized users.

## Background

The need for an effective microbicide to prevent sexual transmission of HIV is a well-understood holy grail in prevention efforts. Beyond effectiveness, other important features of a microbicide include ease of application intravaginally and intrarectally, acceptable product characteristics, affordability, safety with repeated use, thermal and pH stability, and long-lasting effect. A successful microbicide should not disrupt the vaginal flora or mucosal epithelium or alter the pH, which may predispose to additional infections [1]. Other features of more debatable value include non-coital dependence and opportunity for covert use. A microbicide that could dually protect against other sexually transmitted infections or prevent pregnancy may be especially desirable as these conditions increase risk of HIV transmission [2–4].

Copper has known bactericidal, fungicidal, and virucidal properties [5–7]. In vitro, copper-based compounds have been shown to prevent HIV infection through several mechanisms: irreversible inactivation of the essential HIV-1 enzyme protease [8], induction of free radical damage to nucleic acids [9], prevention of p24 production and syncytia formation in HIV-infected cells [9], inhibition of gp120 binding [10, 11], and prevention of viral fusion [11].

Most copper-induced insults to cells are the result of free radical damage to biomolecules [7], which can be countered by eukaryotic cells through mechanisms that sequester, expel, or inactivate the copper ions or efficiently repair the damage [12]. Free copper ions diffusing throughout the vagina have the potential to produce reactive species that could damage the integrity of the vaginal epithelial cell barrier by overwhelming cell repair mechanisms or by interfering with protective microflora [5]. However, copper ions complexed within larger molecules such as sulfonated phthalocyanines (CuPcS) might prevent the production of such reactive species while also precluding absorption across the epithelial barrier and intracellular penetration.

Previous studies have demonstrated that CuPcS both protects against HIV infection of human T cells and elicits HIV inactivation in vitro [9, 11, 13–15]. It has also been shown to be non-toxic against *Lactobacillus* spp., the primary protective vaginal bacteria [14]. Additionally, it prevents cell-associated virus transmission and demonstrates effectiveness in preventing HIV transmission in general throughout a broad pH range, as occurs in the vagina [14]. Although the copper and phthalocyanine groups account for most of the HIV-preventing activity, the sulfate groups may afford additional protection against HIV infection by blocking both the endocytic and receptor-mediated pathways that HIV can utilize to infect cells [16, 17]. The mechanisms by which CuPcS is known to prevent HIV infection include interference with viral envelope attachment to cell receptors and inhibition of viral entry into cells [14]. In a similar way, copper-impregnated filters have been tested in clinical trials to inactivate HIV in breast milk of HIV-positive mothers [15].

This study examines the feasibility of using CuPcS as a novel microbicide. CuPcS has not previously been studied in vivo. Using the established approach for preclinical development of microbicides, the product was tested for toxicity against epithelial cells as well as efficacy in peripheral blood mononuclear cells and TZM-BL cells. Mice and cervical explants provided models for toxicity and efficacy, respectively.

## Results and discussion

**Fig. 1:**
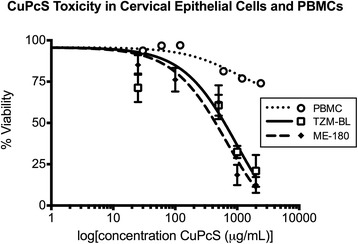
CuPcS Toxicity in Cervical Epithelial Cells and PBMCs. Copper phthalocyanine sulfate (CuPcS) was combined with two cervical epithelial cell lines (ME-180 and TZM-BL) and peripheral blood mononuclear cells (PBMC) at increasing concentrations in quadruplicate. Toxicity was measured as percent viable cells determined by MTT assay or trypan blue exclusion with epithelial cells or PBMCs, respectively, after 48 h of incubation with CuPcS. A dose response effect is observed with increasing concentrations. CC50 values: TZM-BL = 973.7, and ME-180 = 662.6

Toxicity in cell cultures. Nontoxic concentrations of CuPcS were determined by exposing ME-180, TZM-BL, and PBMC cell lines to various concentrations of the compound. CC50 values were determined by applying non-linear regression to the data (Fig. 1). The ME-180 and TZM-BL cell lines demonstrated similar levels of toxicity with CC50s of 662.6 μg/mL and 543 μg/mL, respectively. The CC50 of PBMCs could not be determined as this level of toxicity was never observed. Visual inspection of epithelial cell lines using a light microscope demonstrated slight disruption of epithelial layer integrity at concentrations of 1000 and 2000 μg/mL.

**Fig. 2:**
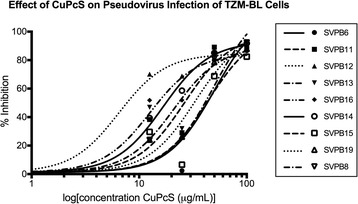
Effect of CuPcS on Pseudovirus Infection of TZM-BL Cells. Copper phtahlocyanine sulfate (CuPcS) was combined with nine different Subtype B, HIV-1 pseudoviruses for one hour prior to addition of TZM-BL cells. Level of infectivity was determined by using a p24 assay performed in triplicate and was observed to have dose–response effect

Antiviral activity in TZM-BL cells. Antiviral activity of CuPcS in TZM-BL cells was determined by pre-treating pseudoviruses with varying nontoxic concentrations of CuPcS and then incubating with TZM-BL cells. A dose–response effect was observed with increasing concentrations of CuPcS (Fig. 2). Non-linear regression models were used to determine EC50 and EC90 values (Table 1). Results were within one order of magnitude across the various pseudovirus strains.

**Table 1:** The EC50 and EC90 values for copper phthalocyanine sulfate (CuPcS) preventing HIV-1 pseudovirus infection of TZM-BL cells were determined with a nonlinear regression model and was comparable across pseudovirus subtypes

Efficacy for pre-treatment of HIV with CuPcS		
	EC50 μg/ml	EC90 μg/ml
SVPB6	50.32	151.6
SVPB11	26.33	79.35
SVPB12	6.467	19.49
SVPB13	21.04	63.41
SVPB16	12.59	37.94
SVPB14	16.67	50.24
SVPB15	43.93	132.4
SVPB19	33.24	100.2
SVPB8	49.92	150.4

**Fig. 3:**
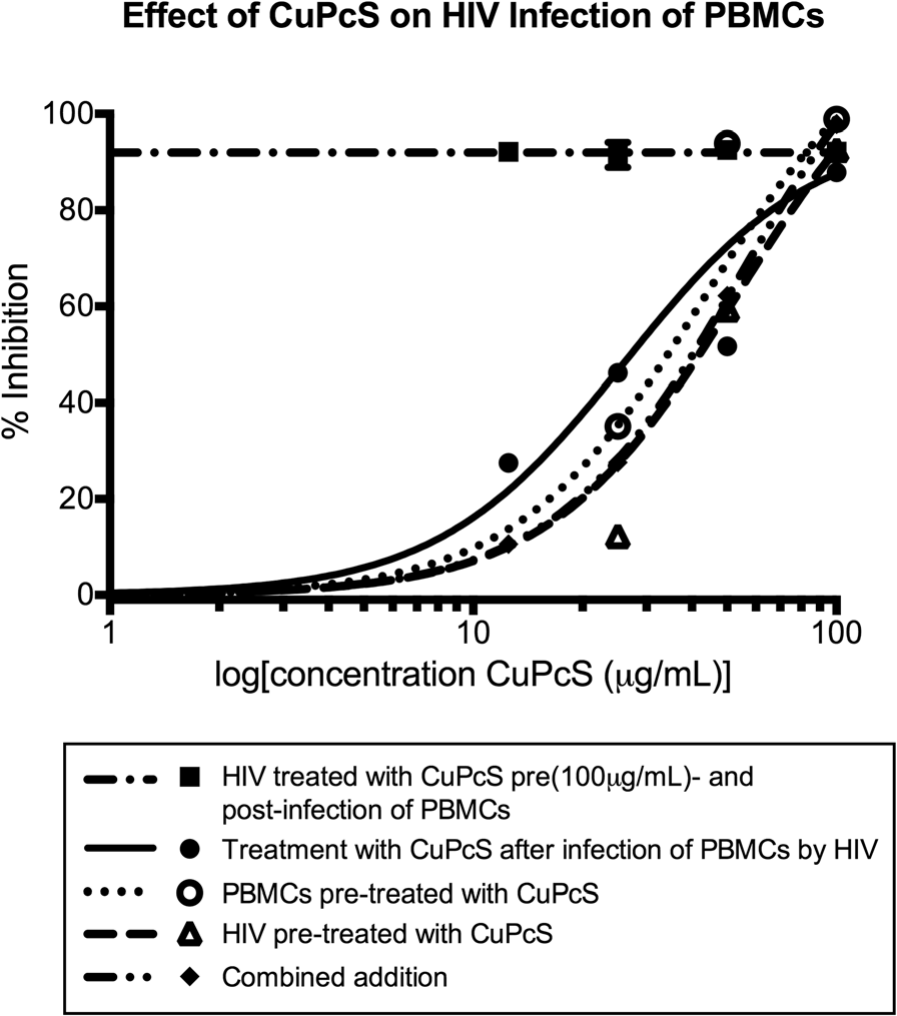
Effect of CuPcS on HIV Infection of PBMCs. A dose–response effect was observed for the prevention of HIV-1_BAL_ infection in peripheral blood mononuclear cells (PBMC) with increasing concentrations of copper phthalocyanine sulfate (CuPcS) under different orders of combination in triplicate

Antiviral activity in PBMCs. PBMCs were combined with HIV-1_BAL_ and nontoxic concentrations of CuPcS. A dose–response effect was observed with increasing concentration (Fig. 3). Non-linear regression models were used to calculate EC50 and EC90 (Table 2). Values were similar regardless of the order of combination except when HIV was pre-treated with 100 μg/mL CuPcS and then combined with PBMCs and additional CuPcS, which was designed to assess irreversible virucidal activity of the compound. Pre-treatment of virus resulted in nearly complete inhibition regardless of post-treatment concentration.

**Table 2:** The concentrations at which 50 and 90 % of infections are prevented under the various conditions of copper phthalocyanine sulfate (CuPcS) exposure: HIV pre-treated with CuPcS; peripheral blood mononuclear cells (PBMC) pre-treated with CuPcS; simultaneous addition of HIV, PBMCs, and CuPcS; and pre-incubation of HIV and PBMCs with subsequent addition of CuPcS

CuPcS Inhibition of HIV-1_BAL_ Infection of PBMCs		
	EC50 μg/ml	EC90 μg/ml
HIV pre-treated	53.31	184.7
PBMCs pre-treated	42.96	147.2
CuPcS added post-infection	26.11	215.3
Combined addition	54.72	186.7
Pre (100 μg/mL)- and post-treatment	Undefined	Undefined

**Fig. 4:**
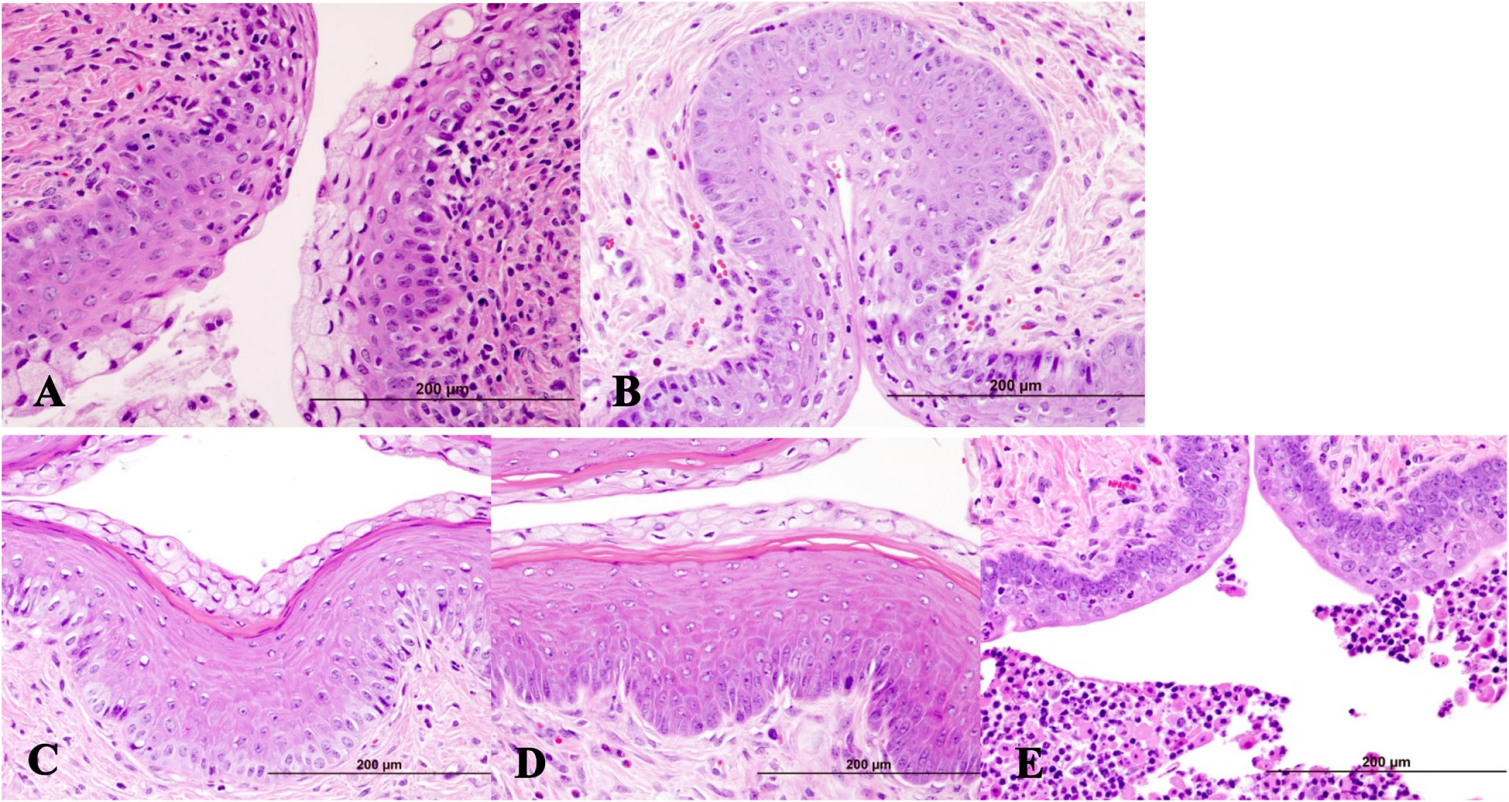
Murine Reproductive Tract Tissue after CuPcS Exposure. The images are cross sections of vaginal tissue samples stained with H&E from mice in various stages of estrus. **a** Mid-vagina: diestrus, Group 2 (1 % Carbopol gel). The stratum germantivum is thickening and the epithelial cells in the superficial layers are demonstrating early mucification. Multifocally, occasional leukocytes are present in the superficial epithelial cell layers and small to small-medium numbers of leukocytes are present in the lamina propria. H&E, 40X. **b** Mid-vagina: diestrus, Group 4 (100 mg/ml CuPcS). The stratum germantivum is thickening and the epithelial cells in the superficial layers are consistent with early mucification. Multifocally, occasional to small numbers of leukocytes are present in the superficial epithelial cell layers and occasional to small-medium numbers of leukocytes are present in the lamina propria. H&E, 40X. **c** Mid-vagina: proestrus, Group 2 (1 % Carbopol gel). The outer stratum mucification layer is composed of epithelial cells containing mucin vacuoles and overlies a thin, eosinophilic stratum corneum. There is an absence of leukocytes in the underlying stratum granulosum and stratum germintivum. Occasional to small numbers of leukocytes are present in the lamina propria. H&E, 40X. **d** Mid-vagina: proestrus, Group 4 (100 mg/ml CuPcS). The outer stratum mucification layer is composed of epithelial cells containing mucin vacuoles and overlies an eosinophilic-staining stratum corneum showing artifactual intra-layer separation of the cornified cells. There is an absence of leukocytes in the underlying stratum granulosum and stratum germintivum. Occasional leukocytes are present in the lamina propria. H&E, 40X. **e** Mid-vagina: diestrus, Group 1 (Control). The stratum germantivum is thickening and the epithelial cells in the superficial layers are becoming polyhedral in shape. Multifocally, small to small-medium numbers of degenerate leukocytes are present in the superficial epithelial cell layers and occasional to small numbers of leukocytes are present in the lamina propria. H&E, 40X

Toxicity in mice. Of the 39 mice evaluated histologically, one of the Group 1 (*N* = 3) control mice, was eliminated due to the presence of bilateral, granulosa cell tumors in the ovary. For the remaining 38 animals, Group 2 (*N* = 12) 1 % Carbopol gel, Group 3 (*N* = 12) 10 mg/ml CuPcS in 2 % Carbopol gel, and Group 4 (*N* = 12) 100 mg/ml CuPcS in 2 % Carbopol gel, standard histologic guidelines were used to determine the stage of estrous prior to evaluating the average number and type of leukocytes in the lamina propria of the uterus and vagina (Additional file 1: Table S1). In the diestrus phase, on average, there were small and small-medium numbers of leukocytes in the lamina propria of the vagina in Groups 3 and 4 (10 mg/ml and 100 mg/ml CuPcS, respectively), and small, small-medium, and medium numbers of leukocytes in the lamina propria of the vagina in Groups 1 and 2 (no CuPcS) (Additional file 2: Table S2). In the proestrus phase, on average, there were occasional, small, and small-medium numbers of leukocytes in the lamina propria of the vagina in Groups 3 and 4 (10 mg/ml and 100 mg/ml CuPcS, respectively), and small and small-medium numbers of leukocytes in the lamina propria of the vagina in Groups 1 and 2 (no CuPcS) (Additional file 3: Table S3). Sample histology photos are demonstrated in Fig. 4. The results strongly suggest that there is no significant difference within or between treatment groups, when segregated by estrous phase, in the level of leukocytic infiltration in the lamina propria of the vagina or uterus when comparing animals treated with 10 mg/ml and 100 mg/ml CuPcS to animals treated with 1 % Carbopol gel alone or with no intervention. However, further studies are needed with larger numbers of animals in each estrous phase to definitively document a lack of statistical significance.

Toxicity in cervical explant tissues. Cervical explant tissues were assayed for toxic effects of CuPcS using a MTT assay. Because the study was performed on tissues previously infected with HIV after exposure to CuPcS, the assay was intended to provide a qualitative assessment of toxicity. The OD/mg tissue remained above the 50 % level. There was no observed loss of tissue viability over the seven days of exposure with increasing concentrations of CuPcS (Additional file 4: Table S4).

**Fig. 5:**
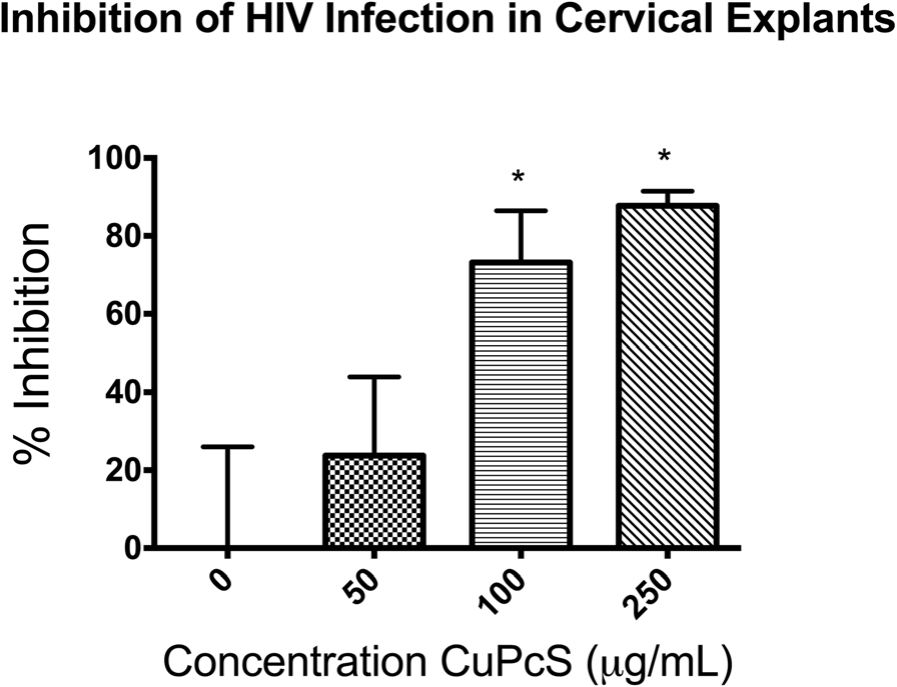
Inhibition of HIV Infection in Cervical Explants. The increasing effectiveness of copper phthalocyanine sulfate (CuPcS) at preventing HIV-1_BAL_ infection of cervical explants with increasing concentrations of CuPcS is presented with standard error bars. Tissues were tested in sets of 4 or 8 at each concentration. Asterisks denote statistically significant differences in inhibition between concentrations of CuPcS using a one-way ANOVA test (*p* < 0.05)

Antiviral activity in cervical explant tissues. Cervical tissue was incubated with CuPcS suspended in cell medium for three hours before the addition of HIV-1_BAL_ and tested for infection at days 3, 5, and 7 with continued exposure to CuPcS. Data from days 5 and 7 were combined as these results were unlikely to contain residual inoculum and would represent cumulative p24 production since the supernatant was changed after each sampling. Using a non-linear regression model, the EC50 for the combined data on days 5 and 7 was determined to be 65.46 μg/mL, and the EC90 was 118.3 μg/mL. Percent inhibition was calculated based on reduction in p24 concentration compared to controls. Statistically significant inhibition of HIV infection was observed for increasing concentrations of CuPcS above 50 μg/mL (*p* < 0.05) (Fig. 5).

The results of the cell-based toxicity experiments reveal CC50 values comparable to other studies with values consistently greater than 500 μg/mL [11, 14]. The in vitro EC50 values are within a log of previously reported values [10, 11, 14]. Reasons for the variability in efficacy may include differences in product purity and preparation, various HIV or pseudo-virus strains tested, and laboratory equipment calibration. This is the first published study to have investigated CuPcS in ex vivo and in vivo models. Previous pre-clinical studies that have shown promise for further development may not have been pursued given an increased focus by researchers and funders on developing microbicides based on known anti-retroviral therapies.

A long-acting microbicide would have sustained contact with the epithelium, potentiating the opportunity for toxic effects to be seen. This study addresses this issue by extending the time of incubation of CuPcS with cell lines to 48 h, observing toxicity to cervical explants over seven days, and exposing mice to daily CuPcS administration for one week. Notably, the lack of observed toxicity occurred despite pre-treating the mice with medroxyprogesterone to enhance their susceptibility to toxic effects by thinning the epithelium.

Toxicity was observed at increased concentrations in the epithelial cell lines, though no gross toxicity was seen at any concentration in mice. This difference may be attributable to the inherent biological protective factors of the vaginal mucosal environment such as a circulating immune response and interplay of vaginal secretions and microflora [18], or it may be a limitation of the study design itself. It is unclear how the in vitro therapeutic index of approximately 100 would translate in vivo as no significant toxicity was seen in non-polarized cervical explants or at concentrations up to 100 mg/mL in the mouse model. However, it should be noted that mouse models are an imperfect representation of the human female genital tract, and further studies using rabbit vaginal irritation models and primates should be performed to better address this issue before carrying out human studies.

Different methods were used to determine the results (MTT assay versus trypan blue exclusion) because the MTT assay requires the addition and removal of solutions at several steps along with subsequent washings to remove the highly pigmented CuPcS that might interfere with the assay. Without adherence of the PBMCs, too many cells were lost to obtain an accurate reading of viability by spectrophotometry. Trypan blue exclusion is often considered an inexact method of assessing viability. However, other studies have demonstrated equivalent results between MTT assays and trypan blue exclusion [18]. Similarly, the results in the present study were comparable among the cell lines regardless of the method used. Though the compound has a prominent blue color at high concentrations, it did not interfere with the MTT assay in controls after adding additional wash steps. One limitation of the data is the assessment of the epithelial integrity. Future studies should include measurements of transepithelial electrical resistance as a means of quantifying epithelial disruption, in particular, because previous sulfate-based microbicides have demonstrated clinical failure as a result of epithelial compromise [19].

A microbicide must also be non-immunogenic in the reproductive tract as immunologic responses to microbicide exposure may recruit more inflammatory cells to the mucosal epithelium, which would provide a larger number of permissive cells at the epithelial barrier at risk of infection [20]. This study showed no increase in the number of leukocytes observed over controls in the reproductive tract tissues of mice.

Susceptible CD4 cells in subcutaneous tissue are considered an important component of transmitting HIV across the epithelial barrier to the blood stream and are primary targets for HIV infection [21]. Thus, preventing infection of these crucial carriers may significantly reduce HIV acquisition. In this study, efficacy in preventing infection of PBMCs produced an expected dose–response effect of CuPcS. With one exception, viral concentrations on day 5 and 6 under the various conditions were nearly equivalent indicating that the order of exposure is not critical. Thus, there may be multiple mechanisms by which CuPcS inhibits HIV infection. The scenario with pre- and post-treatment of HIV resulted in consistent inhibition regardless of the concentration added in the post-treatment step. This may represent irreversible inactivation of the virus by CuPcS because no viral activity returned following removal of the initial compound. Because the preincubation of PBMCs and HIV for one hour prior to CuPcS demonstrated dose–response inhibition, CuPcS may also be acting at a later stage in the viral life cycle. The CuPcS is not anticipated to have interfered with the p24 assay or the viability of the cells because incubation of PBMCs with CuPcS followed by triple washing and the addition of an HIV standard produced the same results as adding that standard to PBMCs alone.

## Conclusions

In this study we demonstrate that CuPcS has activity against HIV-1 in the range of concentrations that are nontoxic to cervical epithelial cells, PBMCs, and cervical explant tissues. Likewise, CuPcS has a broad safety range in cervical explants and the genital tract of mice. This study enhances previous findings by replicating in vitro data and expanding to include both in vivo and ex vivo models [8–11, 13–15]. While these data represent preliminary investigations, the results are promising, and CuPcS should be considered for further evaluation as a potential topical microbicide against HIV.

Useful areas of potential investigation to expand the scope of this study could include the following: measuring effects using other virus clades and cell lines; evaluating for cell-associated virus transmission to see how CuPcS affects the transfer of virus from dendritic cells to PBMCs; conducting more sensitive tests of proinflammatory effects; and performing assays for altered susceptibility, including intrarectally, to subsequent viral challenge in animal models to demonstrate toxicity.

The field of HIV treatment has long battled against the rapid rise in drug resistance. Increasing access to pharmaceuticals in the global population has resulted in greatly expanded life expectancy for those who have been infected with HIV. However, the inconsistent supply of medications coupled with the challenge of adherence to a lifetime of daily drug therapy has caused an increase in primary acquisition and superinfection with resistant viruses worldwide [22–28]. Though partial efficacy to many of the first-line drugs remains, this may pose a mounting challenge to prevention efforts. Thus, it is critical for the field to diversify its prevention portfolios to include innovations such as the one presented here that may interfere at several points of HIV infection and transmission. Furthermore, until there is a prevention modality that meets all the complex needs and demands of the population at risk, researchers and funders should continue to pursue an array of prevention technologies.

## Materials and methods

### Ethics statement

PBMCs were obtained through phlebotomy with consent from healthy, HIV-negative volunteers in accordance with a protocol approved by the Institutional Review Board at the University of Illinois at Chicago (Protocol number: 2009–0737). Ectocervical tissue was obtained with consent from women undergoing hysterectomies for benign disease under a protocol approved by the Institutional Review Board at Rush University Medical Center, Chicago, IL. All research with mice conformed to the Animal Care Policy set forth by the University of Illinois at Chicago Animal Care Committee, which is accredited by the Association for Assessment and Accreditation of Laboratory Animal Care – International, and in accordance with the Guide for the Care and Use of Laboratory Animals of the National Institutes of Health. The protocol was approved by the Office of Animal Care and Institutional Biosafety Committee at the University of Illinois at Chicago (Permit Number: 201101675). Measures to minimize suffering during toxicity studies in the mice included daily observation for signs of mucocutaneous irritation, weight loss, or other evidence of distress, which would be indications to remove the animals from further participation in the study. All animal handlers were trained by the veterinarian of the Animal Care Committee in the careful handing of the animals during administration of the microbicide to place light traction on the back while elevating the hind legs using the tail. Microbicide was administered quickly and gently using new, sterilized, intact pipettes in under five seconds per mouse, minimizing total handling time. This process was determined to be a non-painful and minimally distressing means of microbicide administration by the veterinarian of the Animal Care Committee. Mice were sacrificed using CO_2_ narcosis, which is considered a humane and acceptable method of euthanasia through its painless induction of somnolence.

### Test compound

Copper(II) phthalocyanine-tetrasulfonic acid tetrasodium salt (CuPcS) (molecular weight 988.28) was obtained from Sigma-Aldrich (St. Louis, MO). Stock solutions were created using PBS with a pH of 7.2 at a concentration of 100 μg/mL. This was used for all studies on cell and tissue cultures.

### Cells

Human cervical ME-180 epithelial cells were obtained from ATCC (Manassas, VA, lot no. 57758662) and maintained in McCoy’s 5a medium supplemented with 10 % heat-inactivated fetal bovine serum, 1 % L-glutamine, 100 U/mL penicillin, and 100 μg/mL streptomycin. The TZM-BL cell line, an engineered He-La cervical cell line that expresses CD4, CXCR4, and CCR5 and contains Tat-inducible Luc and ß-Gal reporter genes, was obtained through the NIH AIDS Reagent Program, NIAID, NIH: TZM-BL from Dr. John C. Kappes, Dr. Xiaoyun Wu and Tranzyme Inc. [29], and maintained in Dulbecco’s Modified Eagle Medium (DMEM) supplemented as above with 1 % sodium pyruvate. PBMCs were isolated using gradient-density centrifugation. The cells were cultivated in complete RPMI medium supplemented as with DMEM for three days and stimulated with 10 μg/mL phytohemagglutinin (PHA) and 10 % rIL-2 (obtained through the NIH AIDS Reagent Program, Division of AIDS, NIAID, NIH: Human rIL-2 from Dr. Maurice Gately, Hoffmann - La Roche Inc. [30]). PBMCs were then washed and suspended in complete RPMI with 10 % IL-2. All cells were maintained at 37 °C in 5 % CO_2_.

### HIV-1 stocks

The HIV-1_BAL_, a CCR5 tropic virus, was obtained through the NIH AIDS Reagent Program, NIAID, NIH: HIV-1_BAL_ from Drs. Suzanne Gartner, Mikulas Popovic, and Robert Gallo [31]. Stocks were created by combining the virus with PHA-stimulated PBMCs, adding IL-2, and collecting supernatant at days 5 and 7. Viral concentrations were determined using the p24 Antigen Capture Assay Kit provided by the National Cancer Institute (NIH, Bethesda, MD).

### Pseudovirus preparation

The Standard Reference Panel of Subtype B HIV-1 Env clones was obtained through the AIDS Reagent Program, NIAID, NIH from the following contributors: QH0692 clone 42 (SVPB6), PVO clone 4 (SVPB11), TRO clone 11 (SVPB12), AC10.0 clone 29 (SVPB13) and SC422661 clone B (SVPB8) from Drs. David Montefiori and Feng Gao; pREJO4541 clone 67 (SVPB16), pRHPA4259 clone 7 (SVPB14) from Drs. B.H. Hahn and J.F. Salazar-Gonzalez; pTHRO4156 clone 18 (SVPB15) and pCAAN5342 clone A2 (SVPB19) from Drs. B.H. Hahn and D.L. Kothe [32–34]. The env expression vector pNL4-3.Luc.R-.E- was also obtained through the same NIH AIDS Reagent Program from Dr. Nathaniel Landau.

Stocks of single-round-infection HIV-1 Env pseudovirus were produced by cotransfecting 293 T cell monolayers at 70 % confluency in 6-well plates with 3 μg pNL4-3.Luc.R-.E- and 0.5–4.0 μg env clone plasmid with the transfection reagent polyethylenimine. Pseudovirus stocks were harvested 48 h post-transfection and filtered with a 0.45-μm filter. To test infectivity, TZM-BL cells were inoculated with each of the stocks. Infectivity was determined after 72 h by measurement of chemiluminescence produced by at least a 5-fold increase in luciferase activity relative to the background chemiluminescence of the vector alone. Stock aliquots were stored at −80 °C until use.

### Toxicity in cell culture

Fifty thousand ME-180 or TZM-BL cells were added to each well of a 96-well plate and incubated for 24 h. CuPcS dissolved in PBS was added to the wells with culture medium in quadruplicate to produce final concentrations of 0–2000 μg/mL CuPcS. Cells were incubated with CuPcS for 48 h at 37 °C and then washed with fresh medium. MTT (3–(4,5-dimethylthiazol-2-yl)-2,5-diphenyltetrazolium bromide) Cell Proliferation Assay Kit was obtained from Cayman Chemical (Ann Arbor, MI; lot no. 0417667), and the MTT reagent was added to each well and incubated for 3½ hours. Cells were manually washed with Hank’s balanced salt solution before crystal-dissolving solution was added. The plate was incubated at room temperature in dark conditions for 90 min. Cells were examined using a light microscope for preserved confluence and adherence. Viability was determined through light absorbances read by Vmax Kinetic Microplate Reader (Molecular Devices Corporation, Sunnyvale, CA) at 562 nm. Because of the non-adherence of PBMCs and subsequent difficulty washing out the residual pigmented CuPcS, toxicity in PBMCs was estimated with trypan blue exclusion.

### Antiviral activity in TZM-BL cells

TZM-BL cells were seeded in 96-well plates (5 × 10^3^ cells per well) and incubated at 37 °C in a 5 % CO_2_ atmosphere for 24 h. CuPcS was diluted serially with DMEM and incubated in triplicate with pseudoviruses SVPB6, SVPB11, SVPB12, SVPB13, SVPB16, SVPB14, SVPB15, SVPB19, and SVPB8 (at dilutions equivalent to a 5–15 fold increase in luminescence over background) at 37 °C for 1 h. The mixture was added to TZM-BL cells (100 μL/well), and the plates were further incubated at 37 °C for an additional hour. Then 100 μL DEAE Dextran was added to each well, and the plates were incubated at 37 °C for 48 h. Cells were lysed by adding 100 μL Luciferase Cell Culture Lysis 5x Reagent (Promega, Madison, WI) to each of the wells. Cell extracts were measured for luminescence using Monolight 3010 Luminometer (Pharmingen, San Diego, CA).

### Antiviral activity in PBMCs

HIV-1_BAL_ was grown in PBMCs as described above. Antiviral activity of CuPcS was determined using p24 ELISA. CuPcS was combined with 1,225 pg virus (measured as p24) and 15,000 PBMCs in 96-well plates under various conditions in triplicate: 1) PBMCs were infected with HIV for one hour, then incubated with CuPcS for two hours; 2) virus was pre-treated with CuPcS for one hour before adding PBMCs with or without additional CuPcS; 3) PBMCs were pre-treated with CuPcS for one hour before the addition of virus; and 4) PBMCs, virus, and CuPcS were simultaneously combined. In all conditions, PBMCs were subsequently washed to remove compound and residual virus, and new medium was added. Supernatant was collected on days 4–6 and tested for viral replication. To quantify virus present, p24 concentrations were determined and used to calculate inhibition as percent reduction in p24 concentrations compared with controls.

### Product development

A common polymer used as a microbicide matrix is Carbopol®. Carbopol 940 was selected because of its ability to sustain the viscosity of a thick gel over a broad range of alterations in temperature and pH to aid in retention within the vagina. However, to offset the partial reduction in viscosity posed by increasing concentrations of CuPcS, higher percentages of Carbopol were used for higher concentrations of CuPcS. The final formulations ranged from 1.5–3 % Carbopol with 250 μg/mL – 100 mg/mL CuPcS. These compounds were used exclusively for the studies of toxicity in mice. The compound was neutralized with triethanolamine to a pH around 7.

### Toxicity in mice

Six- to ten-week old female BALB/C mice were obtained from Charles River Laboratories (Frederick, MD). The mice received subcutaneous injections of 3 mg medroxyprogesterone acetate (Greenstone LLC, Peapack, NJ) five days prior to microbicide application. The purpose of the medroxyprogesterone in this study was to mimic the luteal phase of the menstrual cycle by thinning the epithelial lining, thus rendering it more susceptible to potential irritants. Mice received daily intra-vaginal applications of 40 μL of CuPcS in Carbopol using 50-μL Wiretrol glass pipettes (Drummond-Scientific, Broomall, PA) over seven days. Pipettes were manipulated in and out five times to ensure distribution of the microbicide throughout the vagina. Mice were examined daily for signs of genital irritation and then sacrificed with CO_2_ narcosis at 1, 2, or 3 weeks post-microbicide application. Female mouse reproductive tracts were harvested and fixed in 10 % neural buffered formalin for 8–12 h, washed with phosphate buffer solution and stored in 70 % ethanol prior to processing. Sections of ovary, uterus, cervix and vagina were exposed to routine processing, embedded in paraffin and sectioned at 5 microns. Tissue sections were stained with hematoxylin and eosin and evaluated by a veterinary pathologist. Due to the variability of leukocytic infiltrates in the rodent uterus and vagina during the estrous cycle, standard histologic guidelines were used to determine the stage of estrous for each animal [35]. A 7 tier numerical grading scale, i.e., grades 1–7: 1 = occasional (0–3 cells per hpf), 2 = small (4–10 per hpf), 3 = small-medium (11–20 per hpf), 4 = medium (21–30 per hpf), 5 = medium-large (31–40 per hpf), 6 = large (41–50 per hpf) and 7 = marked (>50 per hpf), was used to evaluate the average number of leukocytes in the lamina propria of the uterus and vagina. In randomly selected sites, the number of leukocytes observed in 4–6 fields, were counted at 400X magnification. The numerical field values were then averaged and recorded as a range for within and between group comparisons.

### Antiviral activity in cervical explant tissues

Single, non-polarized pieces of ectocervical tissue approximately 3 mm^3^ in size were placed in inner wells of 48-well culture plates [36]. CuPcS stock solution was diluted using RAFT medium (DMEM containing 24 % Ham’s F-12 medium, 4 mg/mL insulin, 100 Units/mL Pen/Strep, 50 μg/mL gentamicin, 10 % FBS, and 20 mM HEPES buffer). Tissues were incubated with each of the CuPcS concentrations for three hours before inoculation with HIV-1_BAL_ stock at 5,880 TCID_50_. For each condition there were 4 to 8 tissue replicates. Three days following inoculation, tissues were washed twice with RPMI and transferred to a 48-well plate with fresh medium containing appropriate concentrations of CuPcS. Supernatant was removed five hours after the initial wash and replaced with fresh medium. The medium was sampled and exchanged at 5 days post-infection. A third supernatant sample was obtained at day 7 post infection. Supernatant samples were assayed for p24 using the HIV-1 p24 ELISA Kit (catalog # NEK05000, PerkinElmer, Waltham, MA).

### Toxicity in cervical explant tissues

MTT assays were performed on the tissues previously exposed to CuPcS and challenged with HIV to monitor viability. Single explants were added to individual wells of a 48-well plate containing 500 μg/mL of MTT in DMEM in sets of 4 or 8 at each concentration. The explants were incubated at 37 °C for three hours and then transferred to 48-well plates containing 1 mL methanol per well. The plates were incubated at room temperature overnight in the dark. For analysis, explants were blotted to remove excess medium and placed in pre-weighed tubes to determine and record the tissue weight. The absorbance of three replicates of the MTT supernatant fluid was determined at 570 nm on a CERES 900 UV HDi plate reader (Bio-Tek Instruments, Winooski, VT).

### Statistical analysis

The effects of CuPcS on toxicity and efficacy were analyzed using non-linear regression modeling and used to calculate EC50s/90s and CC50s/90s. A two-tailed *t*-test was applied to mean values for percent inhibition in the cervical explant models, and a one-way analysis of variance was applied to assess for statistical significance. The statistical calculations were performed using GraphPad Prism, Version 6.0d (GraphPad Software, San Diego, CA).

## Availability of supporting data

The data supporting the results of this article are included within the article and its additional files.

## Additional files

Additional file 1: Table S1. Number of Animals by Estrous Phase and Treatment Group. The number of female mice in each treatment group is subdivided based on stage of estrus. (DOC 28 kb) Additional file 2: Table S2. Average Numerical Grade of Leukocytes in the Vaginal Lamina Propria during Diestrus by Group. The number of female mice in diestrus is listed according to average leukocyte grade by treatment group. (DOC 43 kb) Additional file 3: Table S3. Average Numerical Grade of Leukocytes in the Vaginal Lamina Propria during Proestrus by Group. The number of female mice in proestrus is listed according to average leukocyte grade by treatment group. (DOC 44 kb) Additional file 4: Table S4. Average Percent Viability of Cervical Explant Cells with Increasing Concentration of CuPcS. Toxicity as measured using MTT assay is presented for cervical explant cells over seven days of exposure to CuPcS. Percent viability was determined as a function of optical density compared with controls. Concentrations were tested in quadruplicate. (DOC 25 kb)
