# Supplementary material for: In vitro antiretroviral activity and in vivo toxicity of the potential topical microbicide copper phthalocyanine sulfate
Source: Virol J. 2015 Aug 30;12:132. doi: 10.1186/s12985-015-0358-5 (PMC4552998; doi:10.1186/s12985-015-0358-5)
Supplement: Additional file 1: Table S1. — Number of Animals by Estrous Phase and Treatment Group. The number of female mice in each treatment group is subdivided based on stage of estrus. (DOC 28 kb) [file 12985_2015_358_MOESM1_ESM.doc]

Supplementary Table 1. Number of Animals by Estrous Phase and Treatment Group

| Number of Mice by Estrous Phase and Treatment Group | | | | |
| --- | --- | --- | --- | --- |
|  | Diestrus | Proestrus | Estrus | Metestrus |
| Group 1 | 1 | 1 | 0 | 0 |
| Group 2 | 2 | 7 | 2 | 1 |
| Group 3 | 7 | 3 | 0 | 2 |
| Group 4 | 5 | 2 | 5 | 0 |
