# Supplementary material for: In vitro antiretroviral activity and in vivo toxicity of the potential topical microbicide copper phthalocyanine sulfate
Source: Virol J. 2015 Aug 30;12:132. doi: 10.1186/s12985-015-0358-5 (PMC4552998; doi:10.1186/s12985-015-0358-5)
Supplement: Additional file 2: Table S2. — Average Numerical Grade of Leukocytes in the Vaginal Lamina Propria during Diestrus by Group. The number of female mice in diestrus is listed according to average leukocyte grade by treatment group. (DOC 43 kb) [file 12985_2015_358_MOESM2_ESM.doc]

Supplementary Table 2. Average Numerical Grade of Leukocytes in the Vaginal Lamina Propria during Diestrus by Group

| Average Numerical Grade of Leukocytes in the Vaginal Lamina Propria during Diestrus by Group | | | | |
| --- | --- | --- | --- | --- |
| Numerical Grading Scale  1-7 | Group 1  Control  N=1 | Group 2  2% Carbopol gel  N=2 | Group 3  10mg/ml Cu in 2% Carbopol gel  N=7 | Group 4  100mg/ml Cu in 2% Carbopol gel  N=5 |
| 1 - Occasional  (0-3 cells) | - | - | - | - |
| 2 - Small  (4-10 cells) | 1 | - | 1 | 1 |
| 3 - Small-medium  (11-20 cells) | - | 1 | 6 | 3 |
| 4 - Medium  (21-30 cells) | - | 1 | - | - |
| 5 - Medium–large  (31-40 cells) | - | - | - | - |
| 6 - Large  (41-50 cells) | - | - | - | - |
| 7 - Marked  (> 50 cells) | - | - | - | - |
