# Supplementary material for: In vitro antiretroviral activity and in vivo toxicity of the potential topical microbicide copper phthalocyanine sulfate
Source: Virol J. 2015 Aug 30;12:132. doi: 10.1186/s12985-015-0358-5 (PMC4552998; doi:10.1186/s12985-015-0358-5)
Supplement: Additional file 4: Table S4. — Average Percent Viability of Cervical Explant Cells with Increasing Concentration of CuPcS. Toxicity as measured using MTT assay is presented for cervical explant cells over seven days of exposure to CuPcS. Percent viability was determined as a function of optical density compared with controls. Concentrations were tested in quadruplicate. (DOC 25 kb) [file 12985_2015_358_MOESM4_ESM.doc]

Supplementary Table 4. Average Percent Viability of Cervical Explant Cells with Increasing Concentration of CuPcS

| Average Percent Viability of Cervical Explant Cells with Increasing Concentration of CuPcS | |
| --- | --- |
| Concentration CuPcS (µg/mL) | Percent Viability |
| 0 | 99.82 |
| 50 | 76.00 |
| 100 | 62.39 |
| 250 | 62.66 |
